# First clinical experience with a new type of albumin dialysis: the HepaWash® system

**DOI:** 10.1186/cc14463

**Published:** 2015-03-16

**Authors:** B Henschel, R Schmid, W Huber

**Affiliations:** 1 TU-München Klinikum rechts der Isar, Munich, Germany

## Introduction

Liver failure (LF) is associated with prolonged hospital stay, increased cost and substantial mortality. With regard to a limited number of donor organs, extracorporeal liver support is an appealing concept to bridge to transplant or to avoid transplant in case of recovery. A new type of albumin dialysis, the HepaWash® system, was recently introduced. The HepaWash® system provides rapid regeneration of toxin-binding albumin by secondary circuits altering binding capacities of albumin by biochemical (changing pH) and physical (changing temperature) modulation of the dialysate.

## Methods

We evaluated the first 14 patients treated with the HepaWash® system with regard to safety and efficacy. Seven patients were treated in the context of the run-in phase of the studies (HEPATICUS 1 and HEPATICUS 2) and seven patients were treated since the HepaWash® system received the CE certificate in July 2013. Patients treated suffered under acute on chronic LF (*n *= 9) or secondary LF which resulted from nonhepatic diseases such as sepsis (*n *= 5). Primary endpoint: comparison of serum bilirubin, creatinine and serum BUN before and after the first treatment with the HepaWash® system. Statistics: IBM SPSS Statistics version 22. The Wilcoxon test for paired samples was used to detect significant treatment effects.

## Results

A total of 254 treatments (1 to 101 per patient) were performed in 14 patients (six female, eight male). Mean age 54 ± 13. MELD score 33.7 ± 7.0, CLIF-SOFA 14.6 ± 2.7. Main underlying disease: nine acuteon-chronic LF; five secondary LF. While bilirubin did not change significantly on the day before HepaWash® treatment (26.2 ± 15.4 vs. 26.0 ± 15.4 mg/dl; *P *= 0.116), serum bilirubin levels were significantly decreased by the HepaWash® procedure (26.0 ± 15.4 vs. 17.7 ± 10.5 mg/ dl; *P *= 0.001). Similarly, serum creatinine (2.2 ± 0.8 vs. 1.6 ± 0.7 mg/dl; *P *= 0.005) and serum BUN (49.4 ± 23.3 vs. 31.1 ± 19.7 mg/dl; *P *= 0.003) were significantly lowered by the HepaWash® procedure. There were no serious adverse events observed in conjunction with the HepaWash® treatment.

## Conclusion

So far the HepaWash® system has proven to be a safe and feasible procedure to effectively eliminate water and protein-bound toxins in humans with LF.

